# Integrated Analysis of the Transcriptome Profile Reveals the Potential Roles Played by Long Noncoding RNAs in Immunotherapy for Sarcoma

**DOI:** 10.3389/fonc.2021.690486

**Published:** 2021-06-11

**Authors:** Boran Pang, Yongqiang Hao

**Affiliations:** ^1^ Shanghai Key Laboratory of Orthopaedic Implants, Department of Orthopaedic Surgery, Shanghai Ninth People’s Hospital, Shanghai Jiao Tong University School of Medicine, Shanghai, China; ^2^ Clinical and Translational Research Center for 3D Printing Technology, Shanghai Ninth People’s Hospital, Shanghai Jiao Tong University School of Medicine, Shanghai, China

**Keywords:** long noncoding RNA (lncRNA), immunotherapy, immune checkpoint inhibitor, resistance, sarcoma

## Abstract

**Background:**

Long-term survival is still low for high-risk patients with soft tissue sarcoma treated with standard management options, including surgery, radiation, and chemotherapy. Immunotherapy is a promising new potential treatment paradigm. However, the application of immune checkpoint inhibitors for the treatment of patients with sarcoma did not yield promising results in a clinical trial. Therefore, there is a considerable need to identify factors that may lead to immune checkpoint inhibitor resistance.

**Methods:**

In this study, we performed a bioinformatic analysis of The Cancer Genome Atlas (TCGA) to detect key long noncoding RNAs (lncRNAs) that were correlated with immune checkpoint inhibitory molecules in sarcoma. The expression levels of these lncRNAs and their correlation with patient prognosis were explored. The upstream long noncoding RNAs were also examined *via* 450K array data from the TCGA. The potential roles of these lncRNAs were further examined *via* KEGG and GO analysis using DAVID online software. Finally, the relationship between these lncRNAs and immune cell infiltration in tumors and their effect on immune checkpoint inhibitors were further explored.

**Results:**

We identified lncRNAs correlated with tumor cell immune evasion in sarcoma. The expression of these lncRNAs was upregulated and correlated with worse prognosis in sarcoma and other human cancer types. Moreover, low DNA methylation occupation of these lncRNA loci was detected. Negative correlations between DNA methylation and lncRNA expression were also found in sarcoma and other human cancer types. KEGG and GO analyses indicated that these lncRNAs correlated with immune evasion and negative regulation of the immune response in sarcoma. Finally, high expression of these lncRNAs correlated with more suppressive immune cell infiltration and reduced sensitivity to immune checkpoint inhibitors in sarcoma and other human cancer types.

**Conclusion:**

Our results suggest that long noncoding RNAs confer immune checkpoint inhibitor resistance in human cancer. Further characterization of these lncRNAs may help to elucidate the mechanisms underlying immune checkpoint inhibitor resistance and uncover a novel therapeutic intervention point for immunotherapy.

## Introduction

Sarcomas represent a cohort of rare and heterogeneous tumors with over 100 different histological subtypes occurring predominantly in the trunk, extremity, and retroperitoneal areas (1). These tumors exhibit a wide range of differing behaviors and underlying molecular pathologies (2–4). Recently, subtype-specific cancer biology has revealed distinct molecular alterations responsible for tumor initiation and progression. The rarity and molecular heterogeneity of sarcoma creates challenges for the development of targeted therapeutics. Fortunately, in contrast to the genomic landscape of other tumor types, the genomic landscape of sarcomas is relatively simple, which offers the opportunity to identify driver events and translate these discoveries into clinically useful biomarkers (5, 6). This may improve the understanding of sarcoma biology, accelerating clinical translational research.

Multidisciplinary management is best for sarcoma therapy (7–12). Surgical resection with or without radiotherapy and chemotherapy is the standard therapy for localized sarcoma (10, 13). Systemic therapy has a prominent role in the multidisciplinary management of locally inoperable or metastatic sarcomas (7, 14). Cytotoxic chemotherapy has been the mainstay therapy for treating advanced-stage disease, with overall response rates of approximately 25% in the first-line setting. In patients with unresectable and metastatic disease, the standard treatment is a single-agent protocol administered with palliative intent. Despite the use of chemotherapy, advanced-stage sarcoma is almost invariably fatal. Thus, it is of urgent importance to develop methods to identify the patients most likely to benefit from current treatments and to develop novel therapeutic strategies. A number of therapeutic targets for sarcoma have been developed with recent scientific technological advancements, including SRC inhibitors, MET and ALK inhibitors, histone deacetylase inhibitors, MDM2 inhibitors and poly (ADP-ribose) polymerase (PARP) inhibitors (15–21). However, these inhibitors yielded unsatisfactory results in clinical trials.

Immunotherapy that has been used for melanoma is a new potential therapy paradigm that holds great promise for sarcoma treatment (22–26). Immune checkpoint molecules target the interface between antigen-presenting cells and T cells and require the clustering of T cells and costimulatory receptors. There are negative-feedback mechanisms to prevent overstimulation, which can be subverted to suppress the immune system in cancer, such as the upregulation of CTLA-4 expression. A significant advance in the understanding of sarcoma immune biology is required as it has recently been shown that the monoclonal antibodies ipilimumab and nivolumab are successful for the treatment of melanoma. The inhibition of CTLA-4 expression with the monoclonal antibody ipilimumab reverses immune suppression and promotes a significant anti-cancer response in melanoma. However, while ipilimumab given to patients with synovial sarcoma was well tolerated, no obvious efficacy was observed (27). Therefore, the identification of potential factors leading to immune checkpoint inhibitor resistance is urgently needed.

## Materials and Methods

### Expression Data and Clinical Data Acquisition

The gene expression profiles and clinical information of patients were obtained from The Cancer Genome Atlas (TCGA) (https://cancergenome.nih.gov/). The clinical data included survival time, survival status, sex, age, TNM stage, pathological stage and so on.

### Identification of Immune Checkpoint Related LncRNA

Immune checkpoint-related lncRNAs were identified through Pearson correlation with immune checkpoint inhibitory molecules (CD80, CD86, CD274, PDCD1 LG2 and LGALS9) (R>0.3 and P < 1.00E-8) according to previous studies (28). The results were analyzed online *via* Venny 2.1.0 software (https://bioinfogp.cnb.csic.es/tools/venny/). P < 0.05 indicated statistical significance.

### Analysis of Prognosis

Overall survival (OS) and progression-free interval (PFI) were defined as the time from treatment until the occurrence of death or progression, respectively. The log-rank test was used to examine the survival difference between different patient groups. P < 0.05 indicated statistical significance.

### Guilt-by-Association Analysis and Functional Enrichment Analysis

Guilt-by-association analysis was performed to identify immune checkpoint-related lncRNAs that are positively correlated with the target genes according to a previous study (29–32). Pairwise Pearson correlation was performed between the expression of immune checkpoint inhibitory molecule-related lncRNAs and all the identified genes. Only positively correlated genes with an R≥ 0.5 and a significant correlation (P < 0.05) were retained. Gene Ontology term enrichment (GO) and Kyoto Encyclopedia of Genes and Genomes (KEGG) pathway analyses were performed using DAVID Functional Annotation Bioinformatics Microarray Analysis (https://david.ncifcrf.gov/) software.

### DNA Methylation Analysis

The DNA methylation profile was measured experimentally using the Illumina Infinium HumanMethylation450 platform from the TCGA. Beta values were derived at Johns Hopkins University and the University of Southern California TCGA genome characterization center. DNA methylation values, described as beta values, were recorded for each array probe in each sample *via* BeadStudio software. DNA methylation beta values are continuous variables between 0 and 1, representing the ratio of the intensity of the methylated bead type to the combined locus intensity. Thus, higher beta values represent higher levels of DNA methylation, and lower beta values represent lower levels of DNA methylation.

### Immune Checkpoint-Related LncRNA Expression and Immune Cell Infiltration

Molecular data for different cancer types and clinical data were downloaded from the TCGA. Moreover, the expression of immune checkpoint-related lncRNAs in different cancer tissues and normal tissues was examined using the TIMER database (https://cistrome.shinyapps.io/timer/). We also reanalyzed immune cell signature genes to estimate the abundance of immune cells (regulatory T cells and myeloid-derived suppressor cells) in the TIMER database. We merged the tumor gene expression with the reference immune cell data of all genes using the Bayes method, as reported in a previous study (33).

### Immune Checkpoint-Related LncRNA Expression and Immune Checkpoint Inhibitor Resistance

Public datasets GSE78220, GSE91061, PRJEB23709, PRJNA482620, and phs000452.v2.p1 from GEO (http://www.ncbi.nlm.nih.gov/geo/GEO), EGA (https://www.ebi.ac.uk/ena/browser/home) and dbGAP (https://www.ncbi.nlm.nih.gov/gap) were downloaded to investigate immune checkpoint-related lncRNA expression and immune checkpoint inhibitor resistance.

## Result

### Identification of Immune Checkpoint Related LncRNA

**Figure 1:**
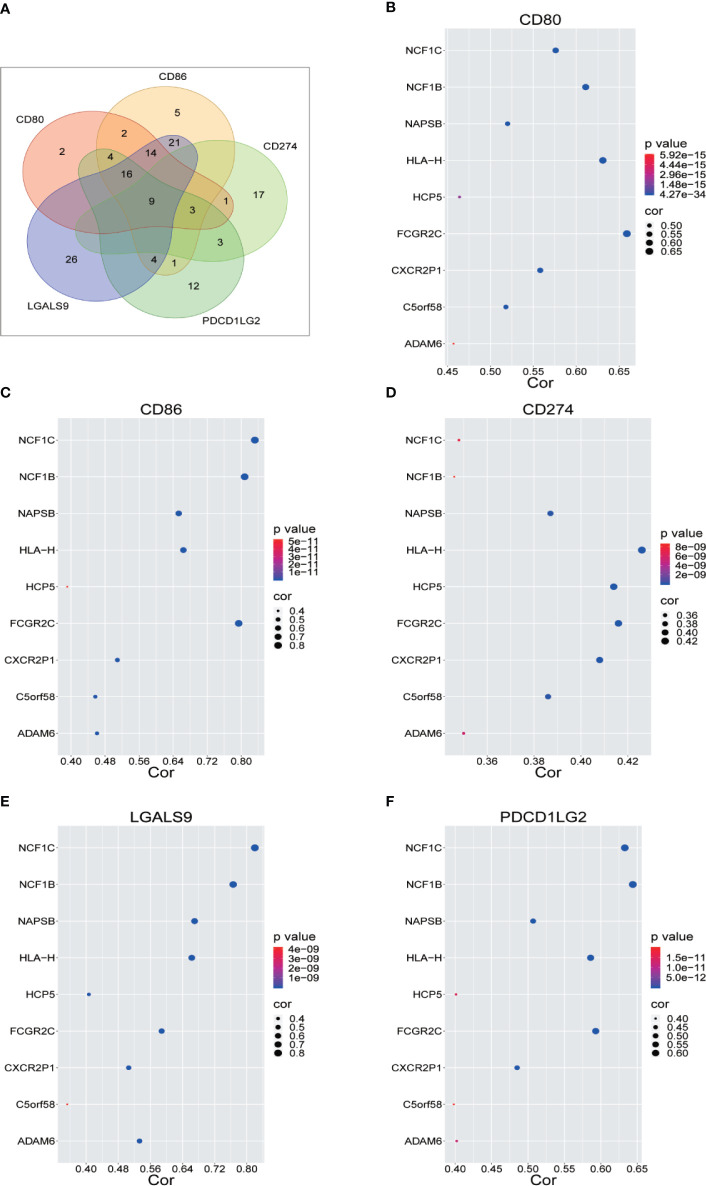
Identification of Immune Checkpoint Related LncRNA. **(A)** Nine lncRNAs were identified through Pearson correlation with immune checkpoint inhibitory molecules, as shown by online Venny 2.1.0 software. **(B–F)** Pearson correlation between nine lncRNAs and five immune checkpoint inhibitory molecules.

We renamed lncRNAs that may promote tumor immune evasion *via* regulation of immune checkpoint inhibitory molecules in human sarcoma “immune checkpoint-related lncRNAs” (ICLs). Sarcoma data from the TCGA were downloaded to analyze the lncRNAs positively correlated with immune checkpoint inhibitory molecules (CD274, CD80, CD86, PDCD1 LG2 and LGALS9), which accelerate the process of tumor cell immune evasion. Nine ICLs (ADAM6, C5orf58, CXCR2P1, FCGR2C, HCP5, HLA-H, NAPSB, NCF1B and NCF1C) that were positively correlated with each of the above inhibitory molecules were identified *via* Pearson correlation analysis (r>0.3, p<5E-09) (**Figure 1A**). The relationship between the identified lncRNAs and each immune checkpoint molecule is shown in detail in **Figures 1B–F**.

### ICLs Were Overexpressed and Correlated With a Worse Prognosis in Human Cancer

**Figure 2:**
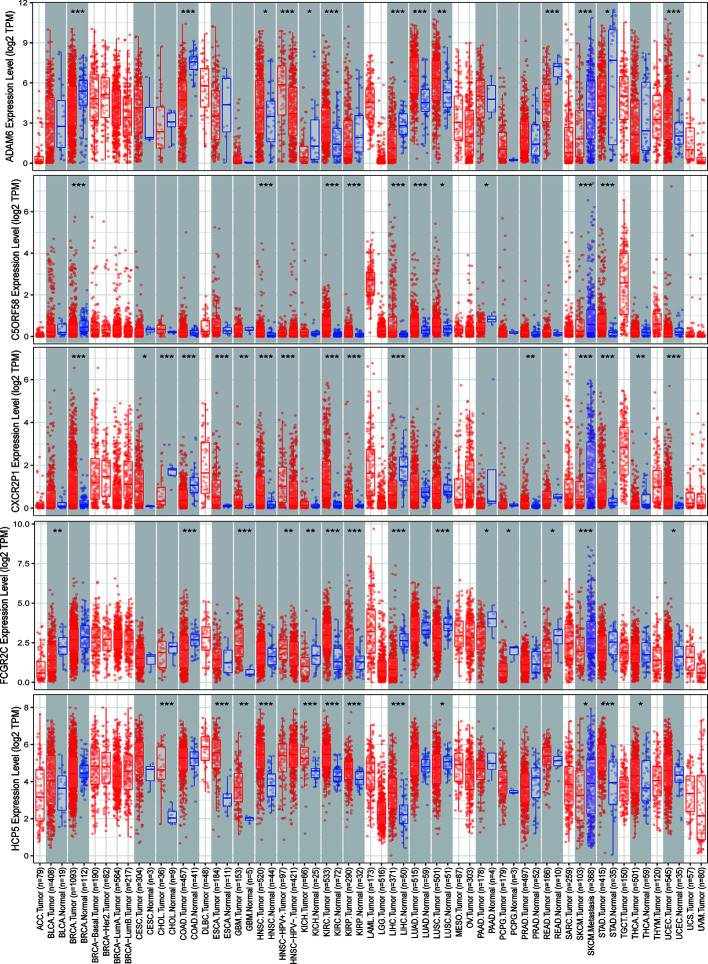
Expression of ADAM6, C5orf58, CXCR2P1, FCGR2C, and HCP5 in different human cancer tissues and adjacent normal tissues. *P < 0.05; **P < 0.01; ***P < 0.001.

**Figure 3:**
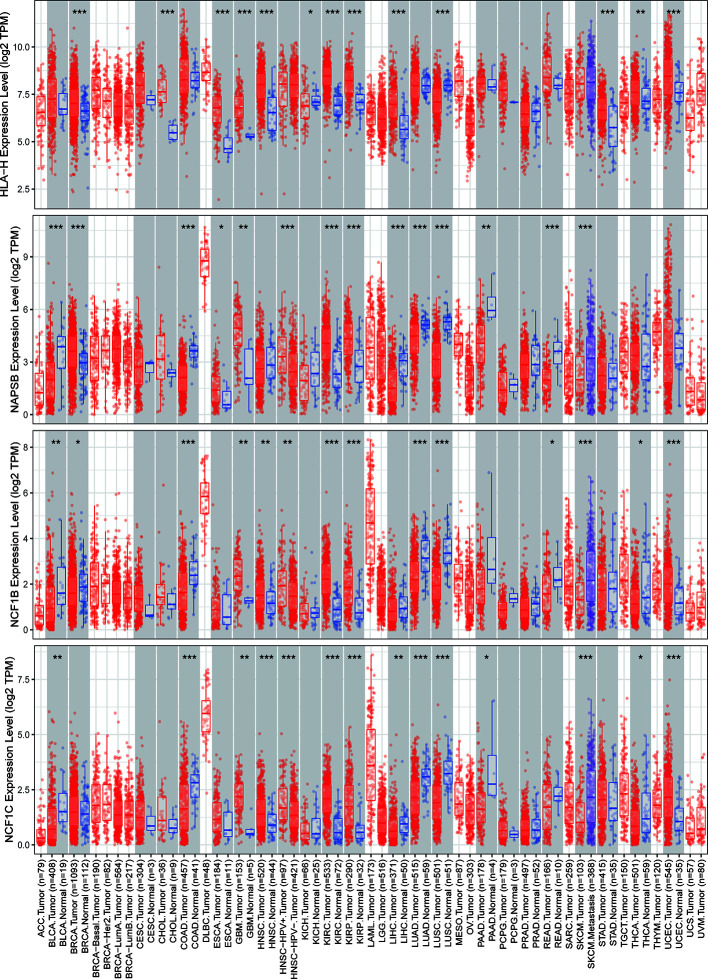
Expression of HLA-H, NAPSB, NCF1B and NCF1C in different human cancer tissues and adjacent normal tissues. *P < 0.05; **P < 0.01; ***P < 0.001.

**Figure 4:**
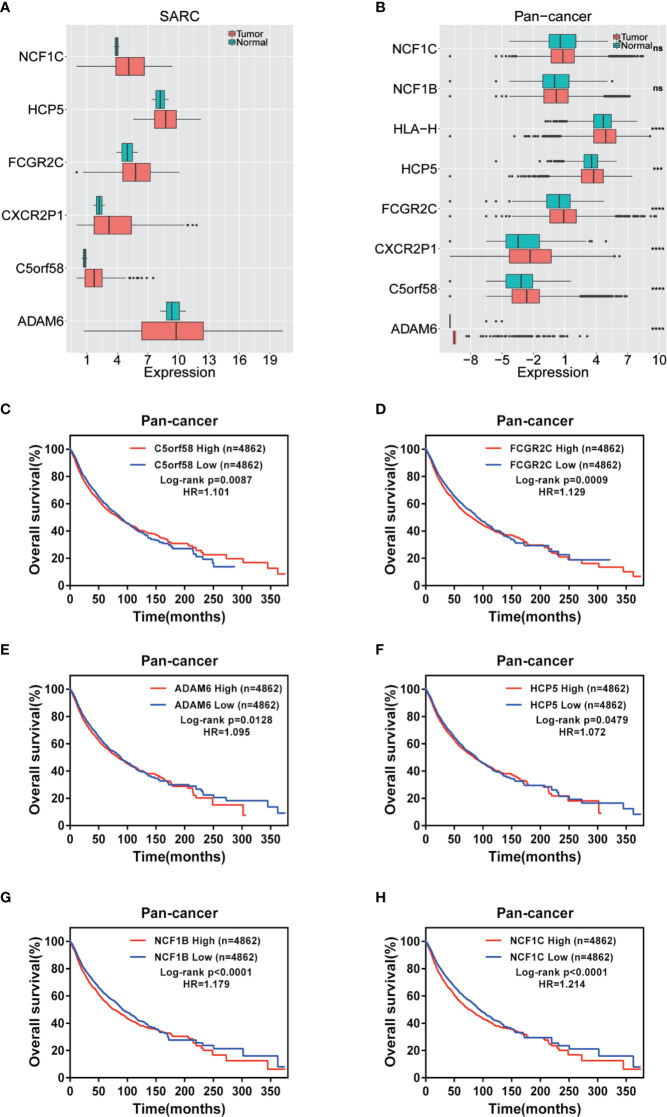
Expression of ICLs and prognosis in human cancer. **(A)** Expression of ICLs in sarcoma. **(B)** Expression of ICLs in pancancer. **(C–H)** Expression of C5orf58/FCGR2C/ADAM6/NCF1B/NCF1C and overall survival across cancers. **(D)** Expression of ICLs and overall survival in pancancer. ***P < 0.001; ****P < 0.0001; NS, No significance.

The expression of nine ICLs, ADAM6, C5orf58, CXCR2P1, FCGR2C, HCP5, HLA-H, NAPSB, NCF1B and NCF1C, was further investigated in different cancer types, including sarcoma. The results showed that the expression of nine ICLs was upregulated in different human cancer types (**Figures 2** and **3**). The expression of nine ICLs was further examined in sarcoma and was upregulated in sarcoma compared with the expression of the ICLs in non-cancer tissues (**Figure 4A**). We obtained a similar result in agreement with that observed in sarcoma in our analysis of the expression of nine ICLs in pan-cancer datasets with 9,724 patients (**Figure 4B**). Furthermore, the relationship between the expression of ICLs and patient prognosis was also examined. The results showed that high expression of C5orf58 indicated worse overall survival in the pan-cancer data (**Figure 4C**). For FCGR2C, ADAM6, HCP5, NCF1B and NCF1C, we obtained a similar result in agreement with C5orf58 (**Figures 4D–H**).

### Low DNA Methylation Modification Leads to Overexpression of ICLs

**Figure 5:**
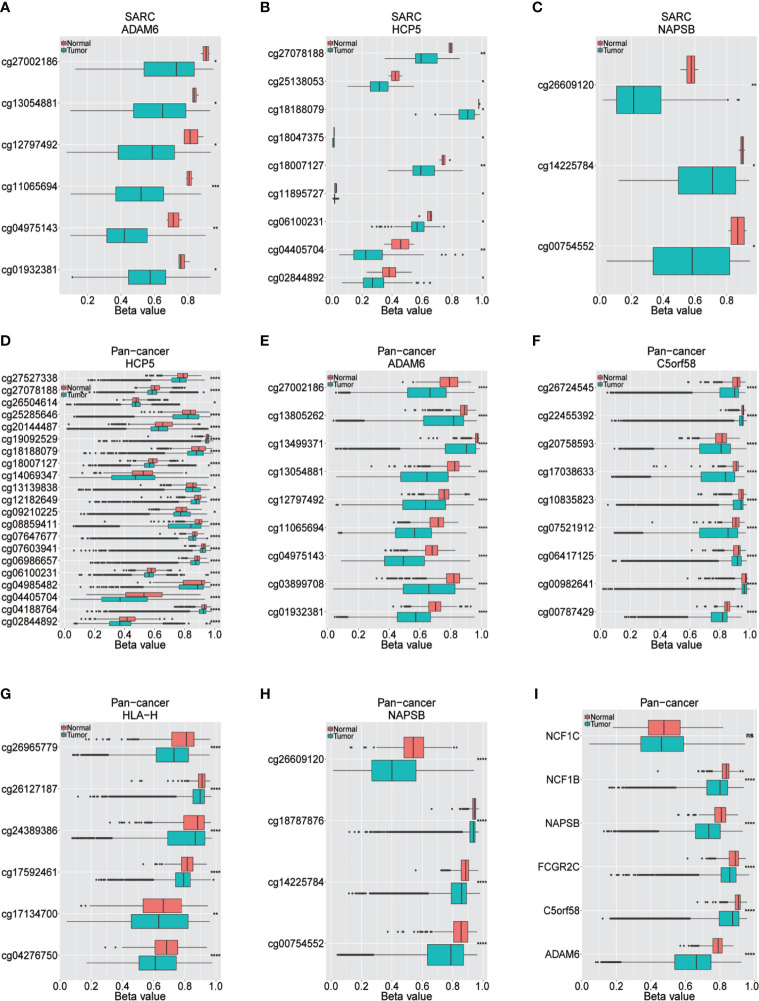
Different methylation sites in ICLs in sarcoma and pancancer. **(A–C)** Different methylation sites in the ADAM6/HCP5/NAPSB/locus in sarcoma tissues and adjacent normal tissues. **(D–H)** Different methylation sites in the HCP5/ADAM6/C5orf58/HLA-H/NAPSB locus in pancancer tissues and adjacent normal tissues. **(I)** Differential methylation of lncRNAs in pancancer tissues and adjacent normal tissues. *P < 0.05; **P < 0.01; ***P < 0.001; ****P < 0.0001; NS, No significance.

**Figure 6:**
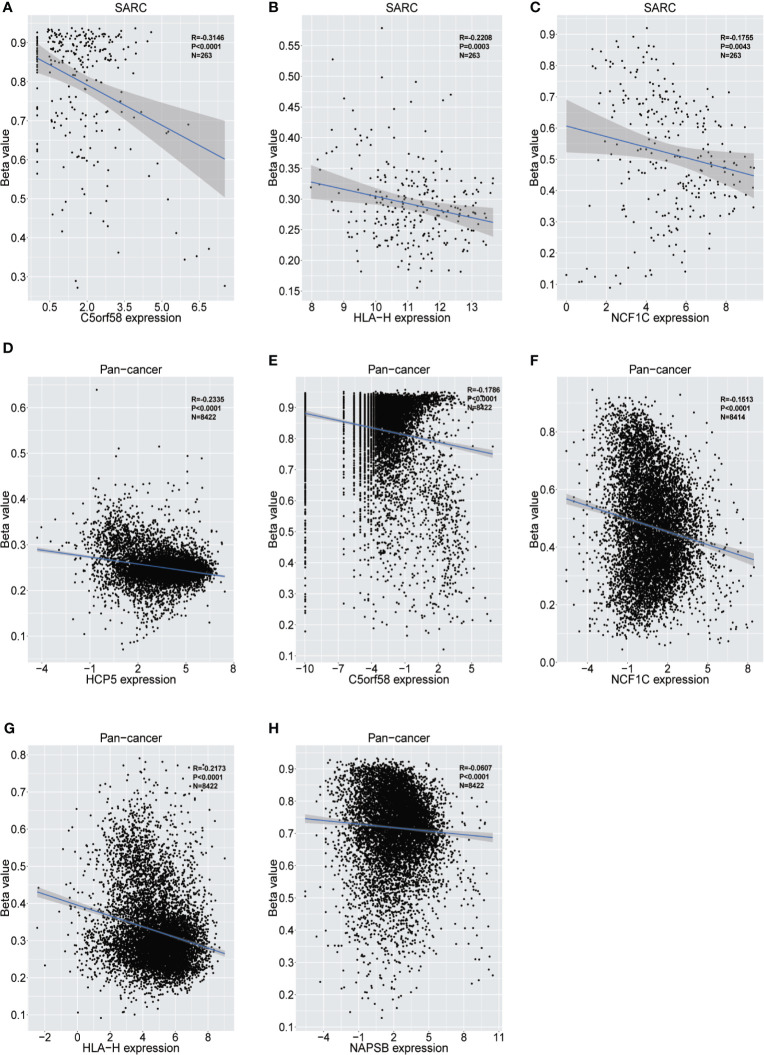
Relationship between the expression of ICLs and DNA methylation in sarcoma and pancancer. **(A–C)** Correlation of C5orf58/HLA-H/NCF1C expression and DNA methylation in sarcoma. **(D–H)** Correlation of HCP5/C5orf58/NCF1C/HLA-H/NCF1B expression and DNA methylation across cancers.

The upstream epigenetic regulation of ICLs was further examined in human cancer. The results showed low DNA methylation at the ADAM6, HCP5 and NAPSB loci in sarcoma cancer tissues compared with that of normal tissues (**Figures 5A–C**). Next, the result was validated in the human pan-cancer data (**Figures 5D–I**). Finally, the relationship between the expression of ICLs and DNA methylation was investigated in sarcoma. For C5orf58, HLA-H and NCF1C, a negative correlation of the expression of ICLs and DNA methylation was observed in sarcoma (**Figures 6A–H**). These results were also validated in the human pan-cancer data.

### ICLs Play Vital Roles in the Negative Regulation of the Immune Response

**Figure 7:**
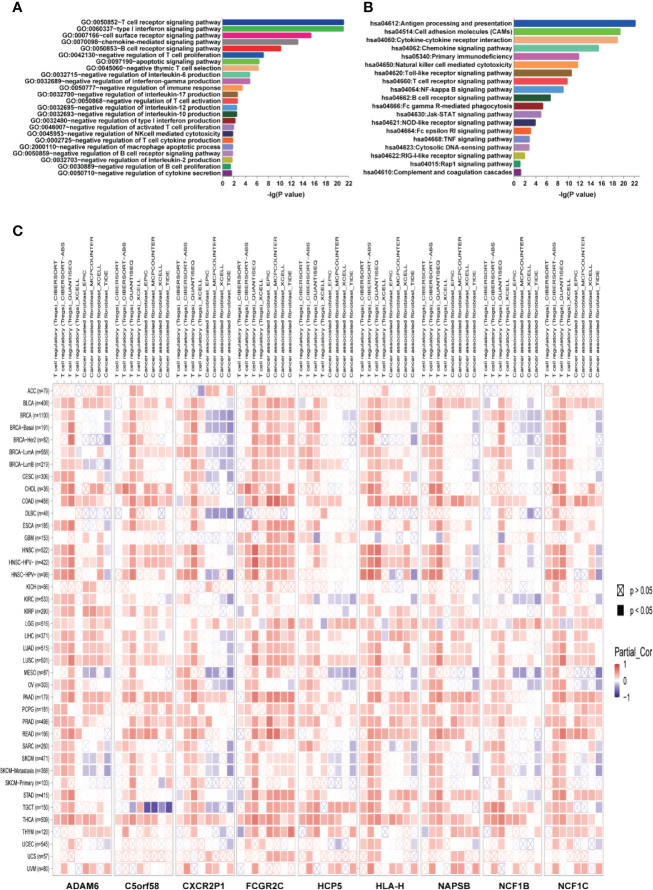
ICLs play vital roles in the negative regulation of the immune response. **(A)** Geneontology term enrichment analysis for ICL-related biological processes. **(B)** Kyoto Encyclopedia of Genes and Genomes pathway analysis for ICL-related pathways. **(C)** Expression of nine ICLs and T cell regulatory/cancer-associated fibroblast infiltration in 33 human cancer types.

The guilt-by-association analysis revealed that the ICLs were involved in the regulation of key immune response processes, such as negative regulation of T cell proliferation, negative thymic T cell selection, negative regulation of interleukin-6, interferon-gamma, interleukin-17, interleukin-12, interleukin-10, type I interferon, interleukin-2 and other T cell cytokine production, negative thymus T cell selection, negative regulation of T cell activation, negative regulation of NK cell-mediated cytotoxicity, negative regulation of the B cell receptor signaling pathway and negative regulation of B cell proliferation (**Figure 7A**). The potential pathways that the ICLs participated in included antigen processing and presentation, chemokine signaling pathway, Toll-like receptor signaling pathway, Rap1 signaling pathway, RIG-I-like receptor signaling pathway, cytosolic DNA-sensing pathway, TNF signaling pathway, Fc epsilon RI signaling pathway, NOD-like receptor signaling pathway, Jak-STAT signaling pathway, Fc gamma R-mediated phagocytosis, B cell receptor signaling pathway, NF-kappa B signaling pathway and T cell receptor signaling pathway (**Figure 7B**). The relationship between ICLs and immune cell infiltration in tumors was further examined in different cancers due to the finding that the ICLs negatively regulate the immune response. As expected, a high expression of ICLs was negatively correlated with T cell regulatory (Treg) infiltration and cancer-associated fibroblast (CAF) infiltration in 33 different cancer types (**Figure 7C**). Tregs and CAFs play key roles in the negative regulation of the immune response, leading to tumor progression due to immune evasion. Thus, the high expression of ICLs may lead to the failure of immunotherapy in human cancer.

### ICLs Confer Immune Checkpoint Inhibitor Resistance in Human Cancer

**Figure 8:**
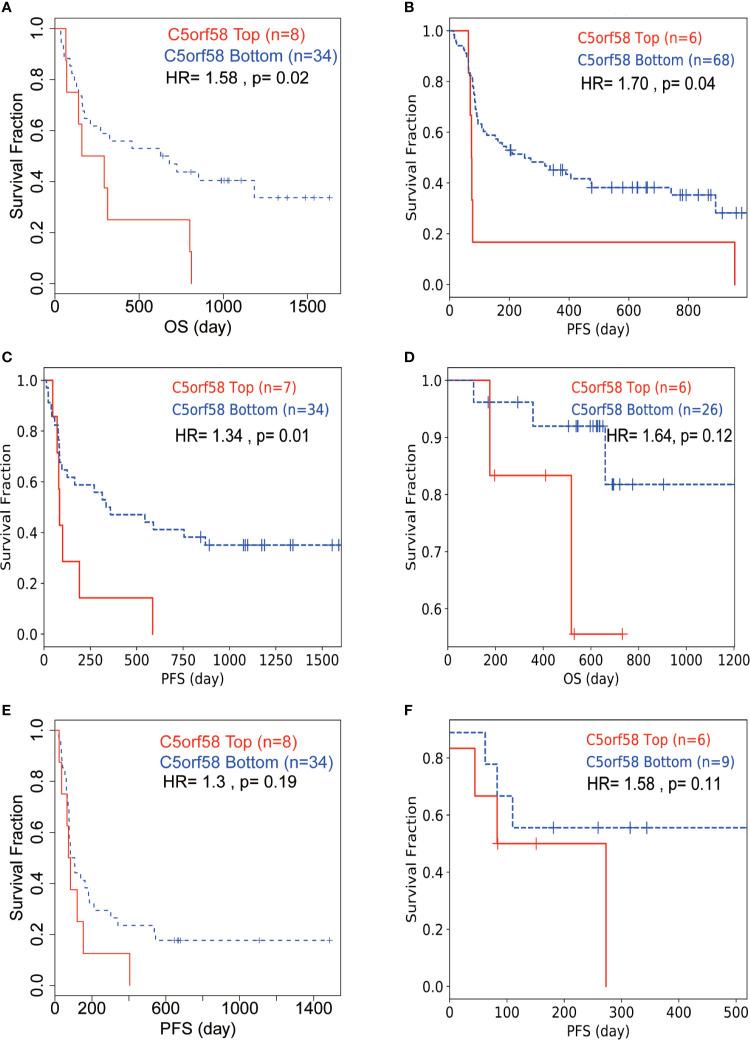
ICLs confer immune checkpoint inhibitor resistance in human cancer. **(A, D, E)** Melanoma patients with high expression of C5orf58 indicated worse response to anti-PD-1/anti-CTLA-4 combined therapy and worse overall survival or progression-free survival in three datasets. **(B)** Melanoma patients with high expression of C5orf58 who received single anti-PD-1 monotherapy showed worse response and worse progression-free survival. **(C)** Melanoma patients with high expression of C5orf58 who received single anti-CTLA-4 monotherapy showed worse response and worse progression-free survival. **(F)** Glioblastoma patients with high expression of C5orf58 who received anti-PD-1 monotherapy showed worse response and worse progression-free survival.

Next, we wondered whether ICLs confer immune checkpoint inhibitor resistance in real-world studies. Public data, including data from patients treated with immune checkpoint inhibitors, were investigated to evaluate the role of ICLs on immune checkpoint inhibitors (34–37). A high expression of C5orf58 in patients with melanoma in different datasets (34, 37) indicated worse response to anti-PD-1/anti-CTLA-4 combined therapy, leading to worse overall survival or progression-free survival (**Figures 8A, D, E**). Moreover, patients with melanoma with high C5orf58 expression who received single anti-PD-1 monotherapy or anti-CTLA-4 monotherapy also showed a worse drug response, resulting in worse progression-free survival (**Figures 8B, C**). In addition, glioblastoma patients with a high expression of C5orf58 exhibited a disappointing response to anti-PD-1 monotherapy and worse progression-free survival (**Figure 8F**).

## Discussion

The success of adoptive cellular therapy for human cancer has resulted in tremendous enthusiasm for immunotherapy (38). Recently, immunotherapy has been approved for different cancers, such as melanoma, prostate cancer, lymphoma, renal cell carcinoma, and breast cancer (39–53). The most successful of these strategies involve immune checkpoint inhibitors. These inhibitors increase endogenous antitumor activity and might increase the tumor immunogenicity that is induced by treatment with chemotherapy, radiotherapy and targeted therapies. These exciting outcomes have led to renewed consideration of the immunotherapy approach for sarcomas.

Studies have indicated decreased overall survival in patients with higher expression of immune checkpoint proteins, such as CTLA-4 and PD-L1, suggesting that blocking immune checkpoint proteins could be therapeutically beneficial (54). The role of targetable immune checkpoint proteins such as CTLA-4 or PD-L1 in sarcomas is not yet well characterized but is a subject of active investigation. A completed phase I trial (NCT00556881) of the anti-CTLA-4 drug ipilimumab in children and adolescents with treatment-resistant cancer included sarcomas, but no results have been reported yet. A second phase II study (NCT00140855) involving ipilimumab for patients with synovial sarcoma was stopped early due to poor accrual and no objective responses. Moreover, another study using the anti-PD-1 antibody SARC028 in patients with bone sarcomas showed a partial response rate. Other inhibitory molecule inhibitors targeting BTLA, LAG-3, TIM3, VISTA, OX40, and CD73 have been developed. However, to date, all clinical trials of immunotherapeutic agents for sarcoma have yielded disappointing results with an insufficient patient immune response.

Primary resistance to immunotherapy may be due to an immunosuppressive environment without a preexisting antitumor response (55). To some extent, immune checkpoint inhibitory molecule overexpression may cause an immunosuppressive environment, leading to immunotherapy resistance in sarcoma. Thus, it is of great importance to identify potential factors leading to immune checkpoint inhibitor resistance. In this study, we identified lncRNAs that might upregulate the expression of immune checkpoint inhibitory molecules in sarcoma. Moreover, these lncRNAs correlated with immune evasion and negative regulation of the immune response in sarcoma. High expression of these lncRNAs correlated with more suppressive immune cell infiltration and worse sensitivity to immune checkpoint inhibitors in human cancer. Thus, this study may indicate a potential cause of immunotherapy resistance issues in sarcoma that requires further investigation. Besides this study, discovery and validation of immune-associated lncRNA biomarkers correlated with immunotherapy have been performed in breast cancer, bladder cancer and lung cancer (56–58). Moreover, loss of MHC or PTEN expression was shown to be another possible mechanism in sarcoma resistance to immunotherapy after an initial positive response (59, 60). Thus, future clinical trials should evaluate the sarcoma tumor microenvironment and the immunological milieu that predicts the immune responsiveness of patients with sarcomas to design more effective immunotherapeutics for sarcoma.

Although initial immunotherapeutic trials for sarcoma yielded disappointing results, rational combinations of immune checkpoint inhibitors with conventional radiation or cytotoxic chemotherapy may substantially improve outcomes for patients with sarcoma in the future. Developing effective options for patients with sarcoma the greatest future challenge. Overall, immunotherapy is a promising strategy, but the current strategies need to be refined for use in patients with sarcoma.

## Data Availability Statement

The original contributions presented in the study are included in the article/supplementary material. Further inquiries can be directed to the corresponding author.

## Author Contributions

YH made substantial contributions to the project conception and design, drafted the manuscript, and gave the final approval of the version to be published. BP and YH analyzed and interpreted the data, drafted the manuscript, and gave the final approval of the version to be published. All authors contributed to the article and approved the submitted version.

## Conflict of Interest

The authors declare that the research was conducted in the absence of any commercial or financial relationships that could be construed as a potential conflict of interest.
